# Hysterical Disorders of Warfare

**Published:** 1919-12

**Authors:** 


### Reviews of Books.

**Hysterical Disorders of Warfare.** By LEWIS R. YEALLAND, M.D. Pp. xii., 252. With Preface by E. FARQUHAR BUZZARD, M.D., F.R.C.P., Lieut.-Col., R.A.M.C. London: Macmillan & Co. Ltd. 1918. Price 7s. 6d. net.

This is a most interesting and remarkable book, both as regards the conception of hysteria and the methods of treatment employed with such success. We cannot help thinking that the personality of the author, his marvellous determination, tact, and good humour, as shown in these pages, is not the least striking of the series of human pictures here detailed. He had studied mental disease in asylum work before turning to the neurological field at the Queen Square Hospital, and the war gave him an unrivalled opportunity of putting his theories to practical use. He has wasted no time in discussing the views which have been taken of the nature of hysteria, but enters at once into the comparison of the symptoms of those disorders due to structural changes and those which are functional or hysterical. With each group of disorders he gives illustrative cases and a minute account of his treatment in each. These forty cases are the most remarkable part of the book. In each we are shown the steps by which he enlisted the patients' co-operation, and by which he more or less rapidly overcame the paralysis tremor or anæsthesia. We are left, however, in doubt how far these records are a verbatim account of what really took place, and if so, how they were taken down, but the conversations go on without a break. The little remarks of the patients, their criticisms and objections, their temporary failures and final success are laid before us in a series of graphic pictures, in which every step is portrayed in the most lifelike manner.

His principle, as Dr. Buzzard tells us in the preface, is based on the belief that a disorder due to suggestion should yield to counter-suggestion. The method of counter-suggestion is immaterial provided only that it is sufficiently forcible. It must be varied with the mental attitude of the patient. A Faradic battery may or may not be used as a means of suggestion, but above all the personality of the medical man must express complete knowledge of his case, untiring determination, tact and decision in every emergency. Practically every case was brought to a successful conclusion at one sitting, though that might require hours, and the patient left the room walking, talking or seeing as well as he had ever done before his illness. We might be disposed to refuse credence to the results of this method, but the number of patients who are known to have been cured both by the author and under Colonel Hurst at Seal Hayne is too great to allow any doubt. The average duration of the functional paralysis or similar disorder before coming under the author's treatment was a year. Exhausted patients, it was found, required some rest before beginning, but when begun the treatment should be carried out without a stop if possible.

With a paralysed or dumb patient, for instance, he impresses upon him that his condition is thoroughly understood, and that he is going to be cured, and that the doctor will not leave until he is cured. He gets the patient to show that he is willing and anxious to be cured, and in a suitable case he explains that there is a negativism or tendency to opposition in the mind which hinders the will being carried out into action. He uses first persuasion, adding Faradism just sufficient to produce a movement in the paralysed limb to demonstrate to the patient that movement is really there, and assures him that the obstruction being overcome, he can hold it in the new position and move it himself as the current is reduced. If he fails to do so the current is increased till his attention is excited and he succeeds. It may be necessary to go on to vigorous compulsion, but usually the patient carries out readily enough his directions, though clumsily and feebly at first as he goes on to order fresh exertions and movements. Frequently antagonistic contractions of other muscles now come on, which he treats by gentle and then increasing Faradism or tires them out by passive movements until the patient can move the limb normally. This takes a long time, but if necessary the patient must continue without interruption the re-education of the movements till he can talk or walk perfectly. The twin principles are persuasion and compulsion, and these must be met by willingness and attention on the part of the patient. Where the willingness is great little compulsion is needful.

He points out the curious fact that in many patients the early stage is one of complete flaccid paralysis. This improves a little under ordinary treatment by massage and Faradism, but rigidity from contraction of antagonistic muscles comes on and is accompanied by pain and sometimes by chronic spasms. Thus rigidity is a less complete stage than flaccidity. Now it is important not to let these patients try to walk till the rigidity has been overcome by vigorous passive movements or Faradism applied to the antagonistic muscles. This is employed as they carry out simple movements in bed, and as soon as the spasm ceases they are drilled in walking properly.

The most complete certainty of diagnosis is necessary lest we mistake organic disease, such as myelitis, for functional conditions, and the author spares no pains in accumulating the diagnostic signs.

We are interested to find that he inclines to the view that hemianæsthesia does exist of itself in many of these patients, and is not due to suggestion by the physician as some authorities lay down. Right hemianæsthesia is curiously often associated with defects of speech.

Two slight misprints occur. On page 105 the words "no such" have dropped out in the sixth line from the bottom and on page 125 the words "of hysterical origin" are needed in the eighth line from the bottom.

